# Burden of caregiving of individuals with GM1 and GM2 gangliosidoses in the United States: a qualitative study

**DOI:** 10.1186/s13023-025-04030-6

**Published:** 2025-11-20

**Authors:** Maria Belen Rodriguez, Ruth Pulikottil-Jacob, Karli Heuer, Nancy Gabriela Perez, Christine Waggoner, Diana Jussila, Chad Gwaltney, Robert Krupnick, Daisy Ng-Mak

**Affiliations:** 1 Sanofi, Cambridge, MA USA; 2 Sanofi, Reading, UK; 3 IQVIA, New York, NY USA; 4 IQVIA, Mexico City, Mexico; 5 Cure GM1 Foundation, Albany, CA USA; 6 National Tay–Sachs and Allied Diseases Association (NTSAD), Boston, MA USA; 7 Gwaltney Consulting, Westerly, RI USA; 8 IQVIA, Boston, MA USA

**Keywords:** GM1 gangliosidosis, GM2 gangliosidoses, Tay–Sachs disease, Sandhoff disease, Caregiver, Burden, Impact, Responsibility

## Abstract

**Background:**

GM1 and GM2 (Tay–Sachs and Sandhoff diseases) gangliosidoses are rare, autosomal recessive, potentially life-threatening, disabling disorders characterized by progressive neurodegeneration, with no disease-modifying treatment. This qualitative study aimed to understand the humanistic burden of GM1 and GM2 gangliosidoses from caregivers’ perspectives by expanding knowledge on the day-to-day responsibilities of primary caregivers and the impacts experienced while providing care and support.

**Methods:**

Focus groups (90-minute duration) were conducted with caregivers (≥ 18 years) under three separate categories based on the age of the individuals with GM1/GM2 gangliosidoses either in-person (attending Annual National Tay–Sachs & Allied Diseases Association [NTSAD] Conference, Colorado, July 2022) or online (recruited through the NTSAD and Cure GM1 Foundation during November–December 2022).

**Results:**

This study included 29 primary caregivers (mean [range] age: 49.0 [37.0–75.0] years) of individuals (children [24.1%], adolescents [31.0%], and adults [44.8%]) diagnosed with juvenile/late-onset GM1 (41.4%) or GM2 (58.6%) gangliosidoses. The caregivers reported that most individuals required mobility aids (64.3%) and experienced speech difficulties (83.3%); they described their caregiving responsibilities as non-stop, pervasive, and often done without additional support, with marginal variance by disease type or patient age. Supporting activities of daily living was the most prominent responsibility (90.0%), followed by symptom/care management (69.0%), ensuring quality of life (45.0%), and maintaining emotional (24.0%) and physical (10.0%) well-being. Caregiving impacted every facet of life; the caregivers reported 25 different impacts, with constant psychological burden (82.8%), physical ailments/strain (62.1%), anxiety/fear/worry (58.6%), financial difficulties (58.6%), limited time for other family members (55.2%), and limitations on relationships outside family (51.7%) having the most significant effects. The caregivers relied mostly on patient advocacy organizations for resources and expressed the need for financial support, broader disease awareness, and disease-modifying treatments. Although providing care and support deleteriously impacted caregivers’ lives, they reported experiencing positive impacts on relationship building, personal development, family cohesion, community support, and life outlook.

**Conclusions:**

This study showed a substantial humanistic burden with long-term impacts among the caregivers of individuals with GM1 and GM2 gangliosidoses. The findings provide important insights to enhance clinical care while advocating for the resources needed to improve caregivers’ and patients’ lives.

**Supplementary Information:**

The online version contains supplementary material available at 10.1186/s13023-025-04030-6.

## Introduction

GM1 gangliosidosis and Tay–Sachs and Sandhoff diseases (collectively known as GM2 gangliosidoses) are rare, autosomal recessive, potentially life-threatening and disabling lysosomal storage disorders. Progressive neurodegeneration occurs as a result of impaired lysosomal breakdown of the glycosphingolipid GM1 or GM2, leading to their accumulation in the brain and spinal cord [1–3]. The estimated prevalence of GM1 gangliosidosis is 1 in 100,000–200,000 live births [4], while that of Tay–Sachs and Sandhoff diseases is 1 in 320,000 [5] and 1 in 380,000 live births, respectively [6].

GM1 and GM2 gangliosidoses present as a disease severity continuum wherein clinical manifestations and experience can vary significantly, particularly between extremes. Progressive deterioration of motor skills including walking, using hands, and speaking or swallowing are a few of the many features observed in individuals with GM1 and GM2 gangliosidoses, potentially imposing increasingly high levels of burden on them and their families, as symptoms and limitations accrue [7–9]. Currently, no proven disease-modifying therapies are available to directly treat these conditions [10], although some manifestations can be treated symptomatically and palliatively. The infantile forms of GM1 and GM2 gangliosidoses are characterized by developmental delays and cognitive impairment, with symptom onset before 6 months of age and are the most severe and fatal forms by early childhood [11, 12]. The late-infantile GM1 gangliosidosis variant has symptom onset around 12–24 months, while the juvenile form onsets after 2 years with symptoms such as unsteady gait, frequent falls, and slurring of speech, followed by ataxia and dysarthria [2]. ; subacute juvenile GM2 gangliosidoses present onset in early childhood with a severe deterioration of motor skills, dysphagia, spasticity, and a decrease in visual acuity [13, 14]. The adult onset forms of both GM1 and GM2 gangliosidoses show attenuated progression with speech impairment, behavioral disorders, clumsiness, muscle weakness, loss of dexterity, ataxia, and dysarthria [2, 14, 15]. Approximately, 40% of individuals with late-onset Tay–Sachs disease develop psychiatric manifestations, including bipolar disorders or psychosis [16–18].

Caregivers are situated at the intersection of their loved ones’ and families’ experiences and the complexities of healthcare systems, emerging as indispensable partners in the management of these conditions. Their unique perspectives can offer valuable information to better understand the multifaceted burden specific to these disorders, although very little is known about caregivers’ experience. Understanding their experience is important for multiple stakeholders, such as clinicians providing healthcare to individuals with GM1 and GM2 gangliosidoses, to optimize advance care planning, quality of life (QoL), and end-of-life care. Investigating caregivers’ experiences can shed light on the factors that contribute to caregiver stress, burnout, and resilience [19], potentially informing interventions aimed at improving the well-being of caregivers and, by extension, the quality of care provided to individuals with the disease [19, 20]. The experience of caregivers is also relevant to policy decision-makers [21, 22], as it can highlight gaps in healthcare services, support systems, and resource allocation for GM1 and GM2 gangliosidoses.

The present study aimed to understand the humanistic burden of GM1 and GM2 gangliosidoses from a caregiver’s perspective by expanding knowledge on the day-to-day responsibilities of primary caregivers while providing care and support to individuals with these diseases across their lifespan and by identifying physical, emotional, financial, and social impacts experienced by the caregivers of individuals with GM1 and GM2 gangliosidoses across different ages.

## Methods

### Study design and participants

This cross-sectional, non-interventional, qualitative research study was conducted in the United States with primary caregivers of individuals diagnosed with GM1 and GM2 gangliosidoses. A primary caregiver was defined as the primary person (≥ 18 years) self-identified or identified by the individual with GM1 or GM2 gangliosidoses providing care, assistance, and support. Participants were identified and recruited through the networks of the National Tay–Sachs & Allied Diseases Association (NTSAD) or the Cure GM1 Foundation.

Semi-structured, 90-minute focus groups were conducted either in-person or online with the caregivers categorized into three groups based on the age of the individuals: children (6–11 years); adolescents (12–17 years); and adults (≥ 18 years) with GM1 or GM2 gangliosidoses. Three in-person focus groups were conducted with the caregivers attending the 44th Annual NTSAD Family Conference in Denver, Colorado during July 2022, while three focus groups were conducted with those contacted and recruited online during November–December 2022.

To facilitate interviews, discussion guides were developed based on iterative, targeted, and manual literature searches on the burden of GM1 and GM2 gangliosidoses, as well as caregiving conceptual frameworks. The discussion guides were developed by researchers with vast experience in qualitative methodologies. The tone of impacts was neutral and included examples of both positive and negative aspects of caregiving. The focus groups explored caregivers’ background, their roles and responsibilities for caregiving, and the impacts of being a caregiver on a wide variety of aspects of their QoL, including physical health, emotional and psychological well-being, social and family relationships, work and career, financial, leisure, and personal goals/plans. In addition, caregivers were asked to describe the resources that were available and utilized for supporting their loved ones, as well as to characterize their unmet needs for caregiving.

This study was approved by the Advarra Institutional Review Board (Columbia, MD, USA). All participants provided informed consent to participate in the study.

### Data analysis

The focus groups were recorded and subsequently transcribed; the interview transcripts were iteratively coded in the MAXQDA qualitative data analysis software (VERBI Software, version 2020) using a combined inductive and deductive approach. An initial codebook was developed based on previously identified concepts and themes; the codebook was further updated as new concepts, and themes emerged and transcripts that had already been coded were again reviewed with the newly added codes. The data were cleaned to ensure the accuracy of frequency counts and general qualitative coding. A saturation analysis was used to determine whether any new concepts emerged in the final focus group. The data were analyzed across the three in-person groups and the three remote groups, in the sequence they were conducted. As no additional concepts were reported after the third of the six focus groups (data not shown), saturation of concepts was likely achieved, and any additional focus groups would be unlikely to elicit new concepts (Supplementary Table 1).

The data from the focus groups were used to develop a conceptual model of the caregiver’s experience providing care and support to the individuals with GM1 or GM2 gangliosidosis. The conceptual model starts with a description of the population of individuals with GM1 and GM2 gangliosidoses, specifically regarding age, gender, disease type, and clinical characteristics. A description of the caregiver population, including age, gender, and relationship to the individual with the disease, was also provided to give context to the group of individuals interviewed, as well as characteristics to take into consideration when understanding this population’s responsibilities and associated impacts. The characteristics of the caregivers and those of their loved ones were hypothesized to directly relate to some of the caregiving impacts.

## Results

### Characteristics of caregivers and their individuals with GM1/GM2 gangliosidoses

A total of 29 caregivers (median [range] age: 47.0 [37.0 − 75.0] years) of individuals diagnosed with GM1 and GM2 gangliosidoses (children [*n* = 7, 24.1%], adolescents [*n* = 9, 31.0%], and adults [*n* = 13, 44.8%]) participated in the study (Table 1). A majority of the caregivers were female (79.3%); those supporting children and adolescents with the disease were not employed and devoted their entire time for their loved ones (71.4% and 77.8%, respectively). All caregivers were relatives (predominantly parents [86.2%]) of their loved ones living with the disease. A majority of the individuals with GM1 and GM2 gangliosidoses (median [range] age: 15.0 [6.0 − 64.0] years) were female (72.4%). Among the individuals living with GM2 gangliosidoses, Tay–Sachs disease was most commonly reported (*n* = 13, 76.5%) (Table 1). The details of the different types of GM1 and GM2 ganglisidoses are further presented in Supplementary Table 2.

**Table 1:** Characteristics of individuals living with GM1/GM2 gangliosidoses and their caregivers

Parameter	Overall (*N* = 29)	Based on the age of the individuals with GM1/GM2 gangliosidoses		
		Children (*N* = 7)	Adolescents (*N* = 9)	Adults (*N* = 13)
**Caregivers**				
**Age in years** ^**a**^				
Mean	49.0	43.0	45.0	56.0
Median [range]	47.0 [37.0–75.0]	42.0 [37.0–49.0]	46.0 [37.0–50.0]	52.0 [38.0–75.0]
**Sex**,** (*n*** **[%])**				
Female	23 (79.3)	7 (100.0)	7 (77.8)	9 (69.2)
Male	6 (20.7)	0	2 (22.2)	4 (30.8)
**Employment status**,** (*n*** **[%])**				
Full-time	6 (20.7)	2 (28.6)	0	4 (30.8)
Part-time	5 (17.2)	0	2 (22.2)	3 (23.1)
Not working	18 (62.1)	5 (71.4)	7 (77.8)	6 (46.1)
**Relationship with the individual with the disease**,** (*n*** **[%])**				
Parent	25 (86.2)	7 (100.0)	9 (100.0)	9 (69.2)
Partner	3 (10.3)	0	0	3 (23.1)
Sibling	1 (3.5)	0	0	1 (7.7)
**Individuals living with GM1 and GM2 gangliosidoses**				
**Age in years** ^**b**^				
Mean	22.0	8.0	14.0	36.0
Median [range]	15.0 [6.0–64.0]	9.0 [6.0–10.0]	14.0 [13.0–16.0]	34.0 [19.0–64.0]
**Sex**,** (*n*** **[%])**				
Female	21 (72.4)	6 (85.7)	5 (55.6)	10 (76.9)
Male	8 (27.6)	1 (14.3)	4 (44.4)	3 (23.1)
**Diagnosis**,** (*n*** **[%])**				
GM1 gangliosidosis	12 (41.4)	3 (42.9)	4 (44.4)	5 (38.5)
GM2 gangliosidoses^c^	17 (58.6)	4 (57.1)	5 (55.6)	8 (61.5)

^a^Unreported: *n* = 2

^b^Unreported: *n* = 1; one caregiver did not report the age of the adult living with the disease

^c^76% Tay–Sachs, 18% Sandhoff, and 6% not reported

*N*, total number of individuals with the disease/caregivers; *n*, number of individuals with the disease/caregivers

**Fig. 1:**
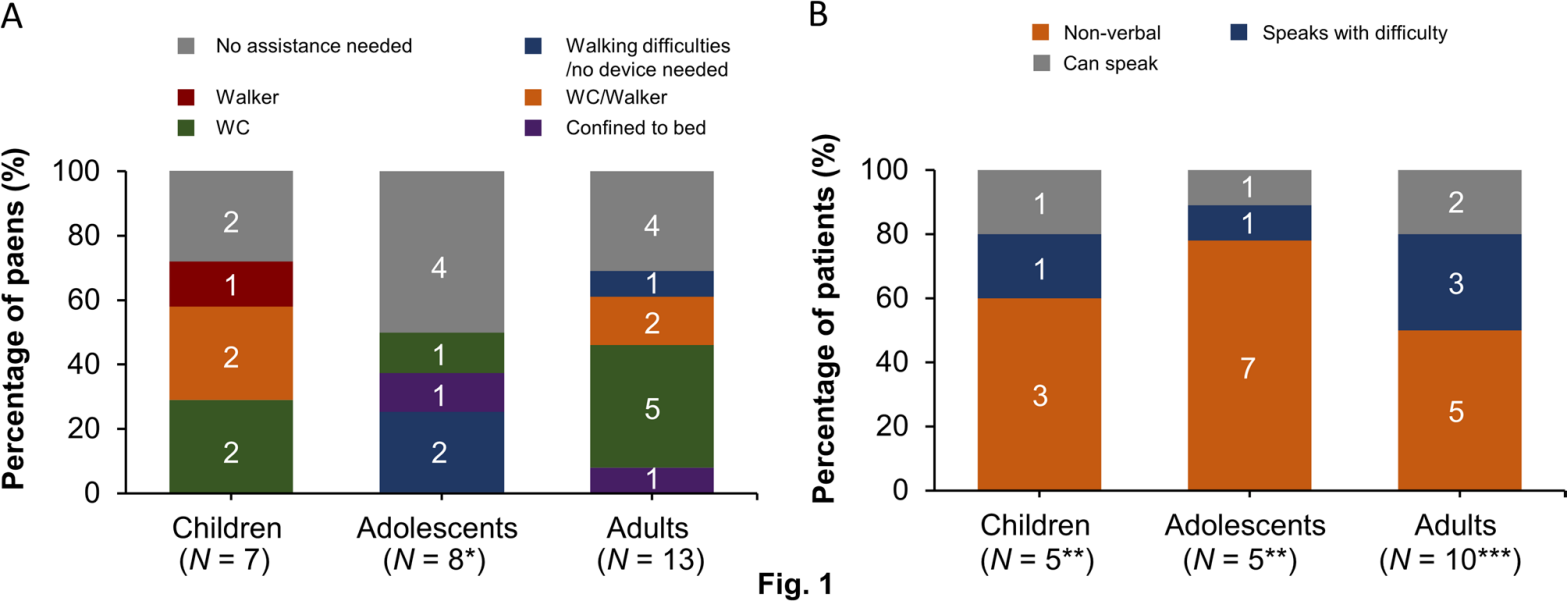
Clinical status of individuals with GM1/GM2 gangliosidoses reported by caregivers: **A** mobility impairment and **B** speech difficulties. The numbers on the bars represent the actual number of individuals with the disease. Questions related to these data were not directly asked to all caregivers because of the nature of focus groups. Unreported data: mobility impairment of adolescents *(*n* = 1); speech difficulty of children **(*n* = 2), and adults ***(*n* = 3). *N*, total number of individuals with the disease; *n*, number of individuals with the disease; WC, wheelchair

The clinical status of individuals with the disease was assessed by considering varying degrees of disease severity concerning mobility and speech difficulties, as reported by their caregivers (Fig. 1). At least half of each cohort of individuals with the disease required mobility assistance (children, 71.4%; adolescents, 50.0%; adults, 69.2%), and more than three-fourths experienced speech difficulties (children and adults, 80.0% each; adolescents, 88.9%) (Fig. 1).

The caregivers further described a diverse number of therapies that they sought for their loved ones to overcome the challenges associated with GM1 or GM2 gangliosidoses. The majority of the children (*n* = 4, 57.1%) received various therapies, such as, feeding, equine, occupational, speech, and physical therapies, as reported by their caregivers. Physical therapy was most commonly reported among adults (*n* = 3, 23.1%), and aquatic therapy was mentioned by one caregiver of an adult (*n* = 1, 0.1%). Among the adolescents, one individual (*n* = 1, 11.1%) received occupational and speech therapies, while another (*n* = 1, 11.1%) received an ‘unspecified’ therapy.

### Caregivers’ responsibilities toward individuals with GM1/GM2 gangliosidoses

Overall, the caregivers reported increased responsibilities and a rapid transition of their role from a “parent” or “partner” to a “caregiver” due to multiple functional losses accrued by their loved ones with disease progression. A caregiver of a child with GM1 gangliosidosis added:

“I think as a parent I care for my daughter in regular ways and even above and beyond ways, however,* there are some things like my daughter has motility issues and is chronically constipated. Every single day I have to give her an enema…That I look at as more of a caretaker. I do it because I’m her parent*,* but I look at that…that’s like an above and beyond caretaker responsibility.”*

On a similar note, a caregiver of an adult with GM1 gangliosidosis explained:

*“So when they were full-time in a wheelchair*,* I was a lot more responsible for getting them from Point A to Point B*,* getting them transferred*,* getting them dressed. When they lost their ability to use the restroom*,* that was then my responsibility. When they lost their ability to eat*,* that was my responsibility. So as milestones started to regress*,* my responsibilities became greater*.*”*

In addition, a caregiver of an adolescent with GM2 gangliosidosis expressed:

*“My son’s caregiving actually started at 4 years old. He was a typical kid until 4. We were just normal parents until he was 4…really fast and it ended up turning into I had to become a caregiver instead of a parent…And it was just drastic. In a couple of weeks*,* I went from a parent to a caregiver and having to learn how to use all this stuff and take care of him*.*”*

The caregivers described their caregiving responsibilities as non-stop, pervasive, and often done alone (i.e., without additional support). This was reported across disease types (GM1 and GM2 gangliosidoses) and age of the individuals with GM1/GM2 gangliosidoses. Moreover, there was a multifaceted nature of caregiving activities as highlighted for example, by a caregiver of an adolescent with GM1 gangliosidosis:

*“My days feel like the same. There’s no weekends off. I’m 24/7. It’s the same thing. There’s no weekends. There’s no time off. I sleep next to her on the floor. I mean, everything’s the same. And then there’s the care, which we have a slightly less severe form, so everything is dragged out longer anyway. So I think how things…right now, all of a sudden we had the throwing up and I’m looking at the G-tube and so I’m trying a hundred different things to make her… But in terms of schedule and how my day goes, it’s like the same day.”*

A caregiver of an adult with GM2 gangliosidosis with a similar experience mentioned:

*“I do all the shopping. I do all the cooking. I do all the cleaning.”*

The caregivers were often reluctant to let others provide care to their loved ones, due to distrust of others’ qualifications and the vulnerability of their loved ones (for example, they were unable to speak and communicate if any inappropriate care was provided). A caregiver of an adolescent with GM1 gangliosidosis stated:

*“…To just have someone that you can depend upon*,* because I’ve had some nurses that I*

*couldn’t. But I think even more so was trust with your child. Especially when you’re dealing with a child who’s non-verbal. If that child were left alone*,* I have to fully trust that you are going to do everything and not harm my child. Because it can be frustrating sometimes.”*

Another caregiver of an adult with GM2 gangliosidosis commented:

*“And you can't trust to leave your children with anybody else either.” *

**Fig. 2:**
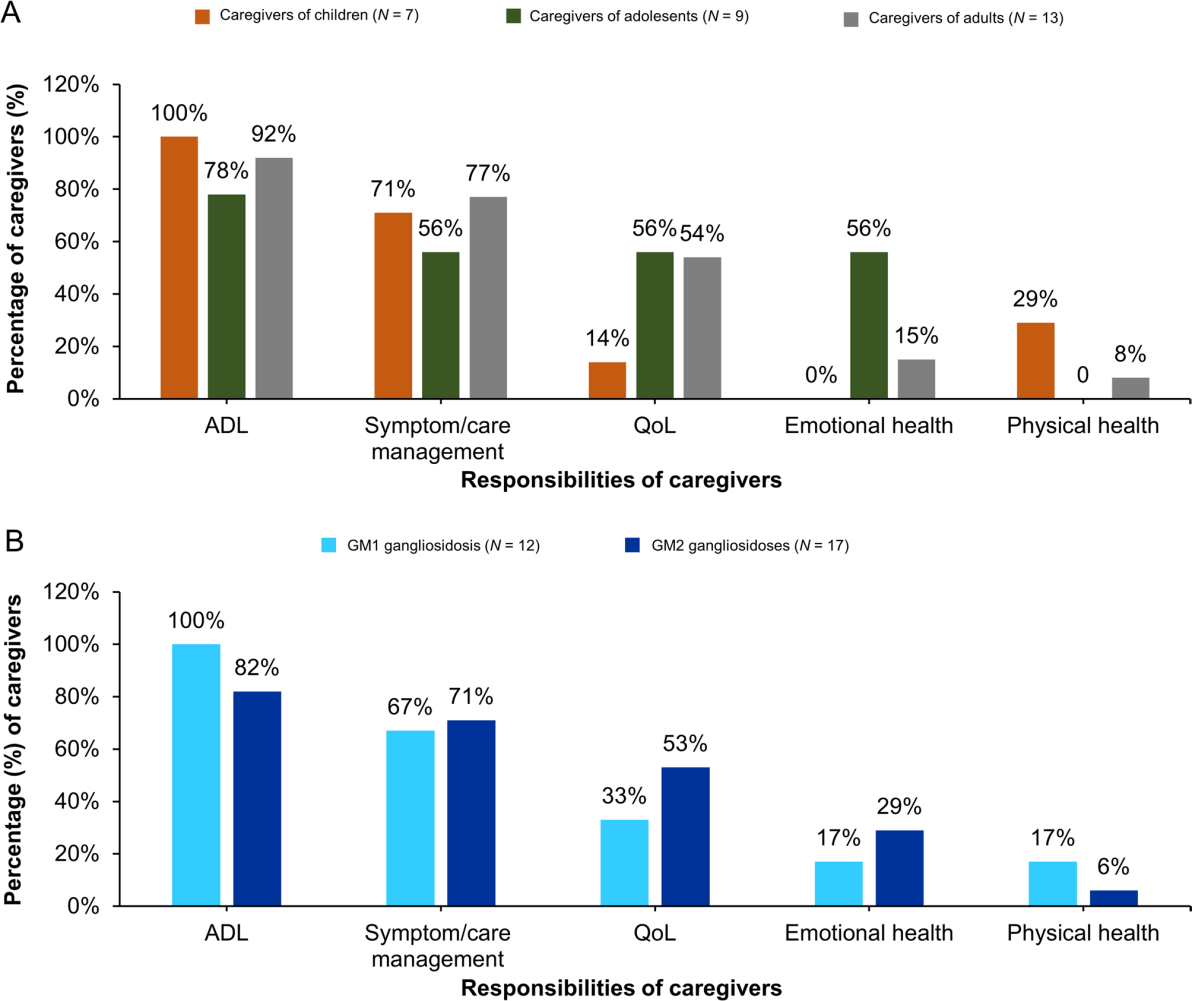
Caregiving responsibilities towards individuals with GM1/GM2 gangliosidoses based on age categories (**A**) and disease type (**B**). Data based on the number of caregivers reporting; due to the nature of a focus group, not all concepts or questions were consistently probed for each caregiver. ADL, activities of daily living; *N*, total number of individuals with the disease in each group; Qol, quality of life

In the analysis of caregivers’ responses, five categories of responsibilities of caregiving emerged as follows: (1) providing assistance with activities of daily living (ADL), (2) managing symptoms and care, (3) maintaining QoL, (4) ensuring physical health, and (5) supporting emotional health (Fig. 2). Overall, minor differences were identified in the distribution of responsibilities across disease types (GM1 and GM2 gangliosidoses), although the study was not designed to compare the caregivers’ burden between the conditions.

The most reported responsibility across caregivers was providing assistance with ADL irrespective of the age of their loved ones living with the disease (children [100%], adolescents [78%], and adults [92%]) (Fig. 2A) and the disease type (GM1 gangliosidosis [100%] and GM2 gangliosidoses [82%]) (Fig. 2B). These responsibilities were significant as individuals with GM1 or GM2 gangliosidoses often required the caregiver to assist with feeding, toileting, dressing, bathing, lifting, and providing mobility support. One of the caregivers of an adult with GM1 gangliosidosis mentioned:

*“You have to care for this person almost like they were a baby or a toddler. I mean, you’re watching out for their safety all day long. You’re bathing this person completely because they cannot bathe themselves. I’m feeding this person because this person can’t feed themselves.”*

A caregiver of an adult with GM2 gangliosidosis with a similar experience mentioned:

*“Laundry, making the bed, changing the bed. Making sure she has meals. It’s like having a baby without having a baby.”*

The second most reported responsibility was symptom care and management across caregivers of children (71%), adolescents (56%), and adults (77%) (Fig. 2A), which included ensuring safety of individuals by preventing falls and choking, monitoring their vital signs, managing medication and doctor’s appointments, as well as scheduling different supportive therapies. A caregiver of an adolescent with GM1 gangliosidosis mentioned:

*“And in a few seconds you see the heart rate goes high and oxygen drop and you don’t know why because everything you do is the same every day. Every medication you give them is the same. Nothing changes, but everything changes.”*

A caregiver whose loved one is an adult with GM2 gangliosidosis expressed:

*“She falls a lot. She’s not a candidate for a walker or what have you because she can’t maneuver them. They’d be more of a detriment to her. She can’t be left alone [anymore].”*

Maintaining QoL in individuals with the disease, such as providing education and/or entertainment, was also a responsibility reported by the majority of caregivers of adolescents (56%) and adults (54%) but only by one caregiver of a child (14%) (Fig. 2A). As stated by a caregiver of an adult with GM1 gangliosidosis:

*“My job is to get her to whatever I think she would want to do to enjoy the day.” *

A caregiver of an adult with GM2 gangliosidosis described:

*“We have I can’t tell you how many get-togethers at our house where everybody comes, and I do all the cooking. I end up doing all the cleaning. But when I can… I only do it for one reason and that’s for her [adult with GM2]. She’s the one that planned it. She’s the one that wanted to have her friends. She’s the one that’s sitting in the pool, enjoying herself, while she’s got this. But for a brief minute, she forgot that she even had it. She forgot that she even has Sandhoff.”*

Responsibilities related to supporting emotional health (24%), including peace of mind, positive attitude, and happiness, as well as physical health (10%), such as providing exercise and other supportive therapies to help maintain muscle strength and function, and ease stiffness, were less prominently mentioned by the caregivers (Fig. 2A). A caregiver of a child with GM1 gangliosidosis described:

“I think parents usually of a child that’s almost [an adult] I think we should not change diapers for her and feed her and get her exercise like a PT something, PT-OT^1^, so we as parents I think usually for a regular child, we just read a story and play games with them, and they’ll be like an interesting joy sometimes, but as a caregiver sometimes it’s frustrating.”

Another caregiver of an adult with GM2 gangliosidosis explained:

*“[REDACTED] was diagnosed in 2015 but as far as a caregiver, I would say probably since. ’14 or ’15. But it wasn’t a physical caregiver as much as it was mentally.”*

Caregivers’ responsibilities across different age groups and disease types are further illustrated in Table 2.

**Table 2:** Caregivers’ perspectives in Understanding their responsibilities towards their loved ones

**1. Activities of daily living**
*Another thing is she was able to chew. I think I just gave her regular food*,* but now I give her pureed food*,* and sort of the tube and more stuff*,* more work to do.* (Caregiver of a child with GM1 gangliosidosis)
*She could never really care for herself in a sense of…she has never been able to dress herself. She’s never been able to go to the bathroom by herself.* (Caregiver of a child with GM2 gangliosidosis)
*His severity is getting rather severe lately. He is completely…tube fed. He can only walk about 20 feet. His muscle tone is deteriorating rapidly. He is non-verbal.* (Caregiver of an adolescent with GM2 gangliosidosis)
**2. Symptom care and management**
*I take her to all of her appointments. We’re out of the house quite a bit going to appointments.* (Caregiver of an adult with GM1 gangliosidosis)
*We have to make sure she doesn’t choke on the foods that we do give her. It has to be blended foods or soft foods.* (Caregiver of a child with GM2 gangliosidosis)
*Because every morning you’re planning for a different circumstance. Which doctor to take him to. Which therapy*,* you have to take him to. Which medications…* (Caregiver of an adolescent with GM2 gangliosidosis)
**3. Maintaining quality of life**
*We just take [REDACTED] with us. She goes out. She’s 31 years old and she goes to bars and she listens to bands. She’s part of a motorcycle group. We try to give her a life. It’s just not 2 of us*,* it’s 3 of us*,* and we tell people that.* (Caregiver of an adult with GM1 gangliosidosis)
*We talk to him as normal people and say hi*,* good morning*,* I’m here. And then during the day all the time I try to give him my love. And then*,* okay*,* I turn the song for him…the song that he had memorized when he was okay. And then*,* okay*,* I sing for you*,* and you can dance with me.* (Caregiver of an adolescent with GM2 gangliosidosis)
**4. Emotional health**
*I oftentimes dream of finding something that I could just give her that would make her happy and content. Because seeing your child in pain hour after hour after hour destroys her…That’s the most unbearable thing for me.* (Caregiver of an adolescent with GM1 gangliosidosis)
*For me*,* the caregiving is to be there more emotionally and keep them in the right frame of mind at this point. It’s hard.* (Caregiver of an adult with GM2 gangliosidosis)
**5. Physical health**
*The stretching I do with my daughter I stretch her on an exercise ball. I also bounce her on an exercise ball to help her relax. It helps her relax*,* and relax her muscles*,* but I also do basically it’s like PT on an exercise ball with my daughter.* (Caregiver of a child with GM1 gangliosidosis)
*So we walk around just to get her some exercise.* (Caregiver of an adult with GM2 gangliosidosis)

Illustrations of each category of responsibility reported by caregivers across different age groups and disease types

### Impacts of GM1/GM2 gangliosidoses on caregivers

The caregivers reported impacts on virtually every facet of their lives, including emotional and psychological, leisure, physical, financial, family, romantic relationships, employment, and personal goals or plans. In total, 25 different impacts were reported by the caregivers (Table 3). The most reported impacts were constant psychological burden (*n* = 24, 82.8%), physical ailments/strain (*n* = 18, 62.1%), anxiety/fear/worry (*n* = 17, 58.6%), financial difficulties (*n* = 17, 58.6%), limited time for other family members (*n* = 16, 55.2%), and limitations on relationships outside family (*n* = 15, 51.7%). For example, a caregiver of an adult with GM1 gangliosidosis spontaneously explained the impact of psychological burden as:

*“The emotions that you mentioned [anxiety, depression, anger, guilt], you wouldn’t be human if you weren’t feeling all those emotions at some point. We all deal with it differently. But somehow, we process through that and we soldier on because we have to soldier on. You have to. You don’t have a choice.”*

**Table 3:** Impacts on caregivers of individuals with GM1/GM2 gangliosidoses

Impacts (%)	Overall (*N* = 29)	Based on age of the individual living with disease			Based on the disease type	
		Children (*N* = 7)	Adolescents (*N* = 9)	Adults (*N* = 13)	GM1 (*N* = 12)	GM2 (*N* = 17)
Constant psychological burden	83	100	78	77	92	76
Physical ailments/strain	62	100	44	54	42	76
Anxiety/fear/worry	59	71	44	62	58	59
Financial difficulties	59	57	44	69	58	59
Limitations on time with other family members	55	57	33	69	42	65
Limitations on relationships outside family	52	86	33	46	58	47
Restriction/ reduction in leisure activities	48	29	56	54	58	41
Stress	41	29	56	38	33	47
Feeling tired/fatigued	38	14	56	38	58	24
Interference with work and employment	38	57	22	38	42	35
Deprioritizing self-care	34	29	33	38	42	29
Frustration	31	71	11	23	25	35
Longing/loss of Opportunities	31	0	33	46	25	35
Interference with sleep	28	14	11	46	17	35
Lifestyle Constraints	24	0	22	38	25	24
Guilt	21	0	11	38	17	24
Sadness/Depression	21	43	33	0	25	18
Grief	17	43	22	0	25	12
Limitations on ability to have romantic relationships	17	29	0	23	25	12
Substance use for coping	17	0	0	38	0	29
Anger	14	0	22	15	8	18
Planning in advance to accommodate medical needs	14	0	22	15	25	6
Disrupting future plans/ goals	7	0	22	0	17	0
Feeling lost	7	0	22	0	8	6
Loss of independence	3	0	0	8	0	6%

Numbers are based on the number of caregivers reporting; due to the nature of the focus group discussion, not all concepts or questions were consistently probed for each caregiver

GM1, GM1 gangliosidosis; GM2, GM2 gangliosidoses; *N*, total number of individuals with the diseases

Another caregiver of a child with GM2 gangliosidosis remarked:

*“It’s hard for me to see the other…the progression of this. It’s tough”*

Similarly a caregiver of an adult with GM1 gangliosidosis experiencing financial impact mentioned:

“I left my job in 2000, so it’s been 22 years, to be a full-time caregiver to the girls. That also impacted our finances.”

A caregiver of an adult with GM2 gangliosidosis spontaneously mentioned:

“There is a financial responsibility. What’s going to happen with her when I’m not around? But it’s not only financial. It’s who [will care for her].”

Broad impact categories with quotes from caregivers describing the nature of these impacts are listed in Table 4. Regarding the age of the person living with the disease, the distribution of impacts varied slightly, leading to constant psychological burden and physical strain on all caregivers of children and adults and mild-to-moderate strain on the caregivers of adolescents (Table 3). The impact of caregiving was mostly reported in psychological well-being, irrespective of the disease type (GM1 and GM2 gangliosidoses, 92.0% and 76.0%, respectively) (Table 3).

**Table 4:** Categories of impact on caregivers with illustrations

**Emotional: Psychological burden, depression, anxiety, fear, sadness, worry, frustration**
*And I feel overwhelming all the time. For me*,* sometimes I cry. I need to drain my feelings because I feel like*,* oh my gosh*,* how to help my daughter.* (Caregiver of an adolescent with GM1 gangliosidosis)
*…it’s hard because I need to take care of her every day*,* and I need to think about her future*,* and I also need to deal with the teachers*,* therapists*,* and the doctors*,* medications*,* everything*,* so sometimes it’s really*,* yeah*,* frustrating.* (Caregiver of a child with GM1 gangliosidosis)
*…we were decorating our Christmas tree and pulled out an ornament where our daughter*,* she used to be able to write her name. So there’s an ornament where she hand-wrote her name. That grief of that loss of her not being able to do that overtook me at that time. So it’s that constant state of this loss.* (Caregiver of an adolescent with GM2 gangliosidosis)
*I think there’s a lot of things that are constant*,* every day*,* all day long that we all worry about. It’s different for each one of us. But then there’s also some new things that go from day-to- day because of what we have going on in our life. That might sound*,* “Okay*,* I got these 10 things and today*,* oh*,* I’ve got these 3 more that we have to worry about.” So it’s a constant…* (Caregiver of an adult with GM2 gangliosidosis)
**Physical: Physical strain on body due to lifting**,** assisting individual with disease**
*It’s a very physically demanding job and it’s a lot of wear and tear…I have shoulder issues. I’ve got back issues. I’ve got knee issues. My husband’s also dealing with back issues.* (Caregiver of an adult with GM1 gangliosidosis)
*And then physically exhausting because you’re having to move your child multiple times a day. Moving them from one piece of equipment to another. Or moving them from one room to another. Or into a bath.* (Caregiver of an adolescent with GM2 gangliosidosis)
**Leisure: Unable to participate in activities**,** no opportunity to enjoy**,** pursue hobbies**
*The word “leisure*,*” that is not within our vocabulary. That has been erased from our vocabulary.* (Caregiver of an adolescent with GM1 gangliosidosis)
*Yeah*,* going out of the house becomes too much of an event. Or it can be too overwhelming. Or you’re too worried about what could happen*,* what could go wrong. So going out and trying to do something leisurely just becomes non-existent just because it’s too big of an ordeal and too much worry.* (Caregiver of an adolescent with GM2 gangliosidosis)
**Family life: Less time to spend with family**,** strain on relationships**
*I think*,* yeah*,* this definitely doesn’t have a good impact to the family relationships too.* (Caregiver of a child with GM1 gangliosidosis)
*I mean*,* ultimately*,* I got divorced mostly because of her diagnosis. No fault to [him]*,* just we handled things differently. He shut down and I didn’t*,* so we got divorced.* (Caregiver of an adult with GM2 gangliosidosis)
**Financial: Loss of income**,** insurance challenges**,** cost of medications/equipment**
*…not only am I spending a lot of my energy doing caregiving*,* but I’m spending a lot of my energy battling for the things [financial assistance] that I need to do the caregiving.* (Caregiver of an adult with GM1 gangliosidosis)
*I have to work. I mean in general*,* in order to support her lifestyle and my lifestyle. I am 73 years old and I work full-time.* (Caregiver of an adult with GM2 gangliosidosis)
**Personal goals/plans: Unable to pursue retirement plans**,** cannot travel/live the life imagined**
*Yeah*,* so we don’t go out*,* and we don’t get any vacation.* (Caregiver of a child with GM1 gangliosidosis)
*When I retire at 65*,* I’m not going to have that typical retired life where it’s time for me and my wife to go and travel and do.* (Caregiver of an adult with GM2 gangliosidosis)
**Work/career: Leaving career**
*I’ve had a job since I was like 16 years old*,* so I’ve always loved working. I’ve always found I’ve been very fulfilled by that. It’s hard [to give it up].* (Caregiver of a child with GM1 gangliosidosis)
**Social life: No time for friends**
*I feel completely cut off from society. It’s near impossible for us to participate in anything that goes on in our community. Or to go to a restaurant or to meet with friends or to have vacations.* (Caregiver of an adult with GM1 gangliosidosis)
*We suffered*,* many of our relations were drift away after the diagnosis.* (Caregiver of an adolescent with GM2 gangliosidosis)

The caregivers had an enormous sense of responsibility toward their loved ones and managed the impacts of caregiving to continue with their caregiving activities. A caregiver of an adolescent with GM2 gangliosidosis mentioned:

“The emotional fatigue that you feel, but yet you’ve got to keep that in check because you have a job to do. So I think that emotional fatigue builds up because you just have to move on through your day to get through.”

Another caregiver of a child with GM1 gangliosidosis described:

“But I feel just for some people ’it’s just hard to process. ’It’s difficult as a parent when you need to just get to a point, especially after a couple of years of diagnosis that you feel like the parent needs to sort of just get over it and step up, face what reality is.”

The caregivers were requested to choose the top three impacts in their life from the eight categories (emotional, physical, leisure, family life, financial, personal goals and plans, work/career, and social) of broad impacts identified. Emotional, physical, and leisure impacts were the top three bothersome impact categories described by overall caregivers (Supplementary Fig. 1). The top three impact categories for the caregivers of children were emotional (85.7%, *n* = 6), followed by family life, and leisure (42.9%, *n* = 3 each); these categories for the caregivers of adolescents were physical (66.7%, *n* = 6), leisure (55.6%, *n* = 5), and emotional tied with personal goals and plans (33.3%, *n* = 3 each), while those for the caregivers of adults were emotional (69.2%, *n* = 9), physical (61.5%, *n* = 8), and family life (46.2%, *n* = 6) (Supplementary Fig. 1).

### Positive revelations of caregivers

**Fig. 3:**
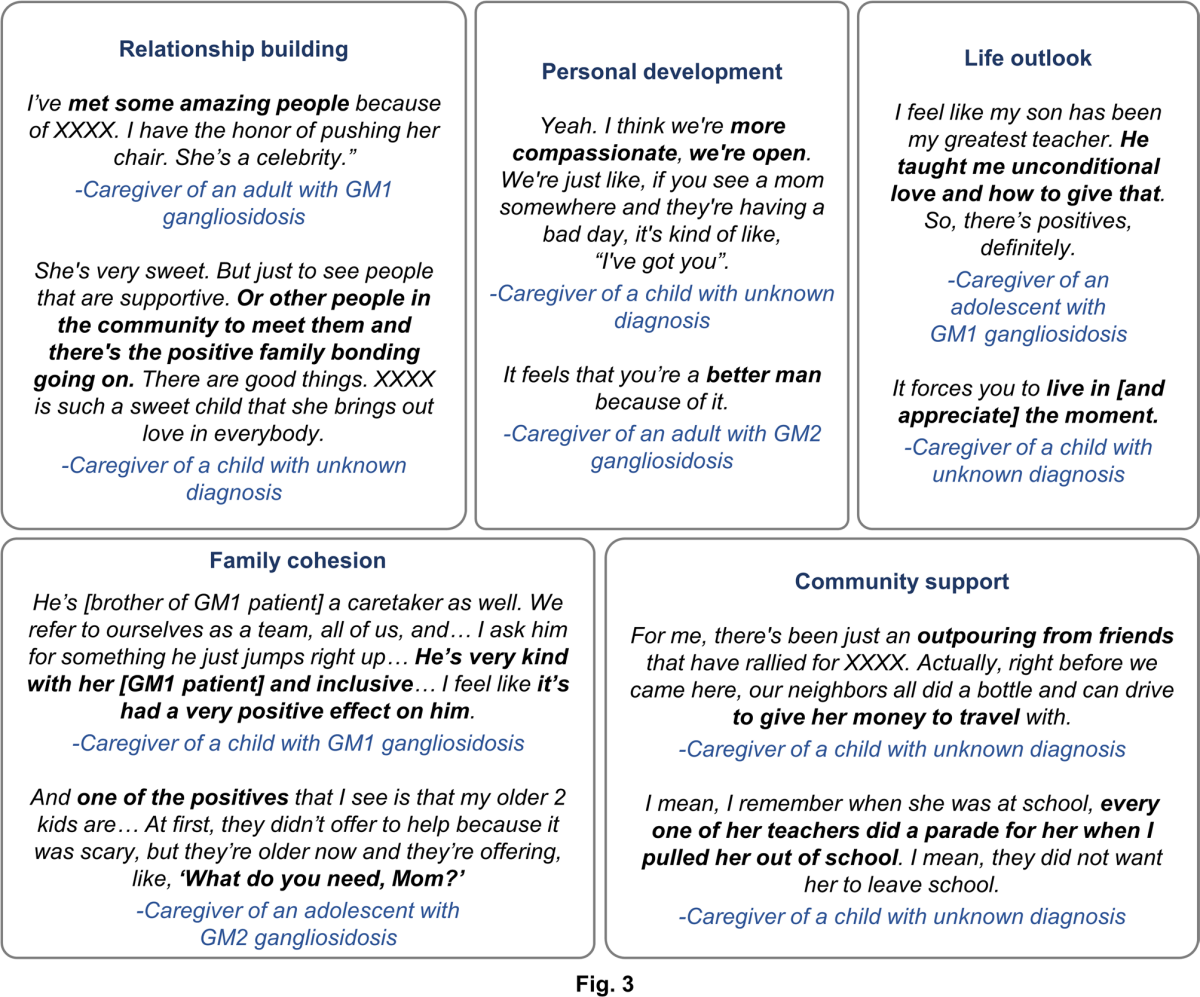
Positive impacts experienced while caregiving individuals with GM1/GM2 gangliosidoses. For the child with an unknown diagnosis, attributing the quote to a specific participant was not possible given the qualitative nature of the study

The caregivers identified some “positive” impacts on their lives in the course of providing care, assistance, and support. These revelations were categorized as: relationship building, personal development, family cohesion, community support, and life outlook (Fig. 3). Some caregivers reported building relationships with people in the GM1 and GM2 gangliosidoses communities, which provided a vital support system and a reliable source for best practices to care for individuals living with these diseases. While caring for their loved ones, the caregivers reported a positive personal development, which improved them as a person and their self-image. The caregivers acknowledged that their roles changed their attitude toward living their life in a positive way. The caregivers also reported receiving family support in several ways, thereby increasing emotional connection; they also referenced broader community support, including financial resources and emotional support from other members of their communities over time.

### Resources available for caregiving

**Fig. 4:**
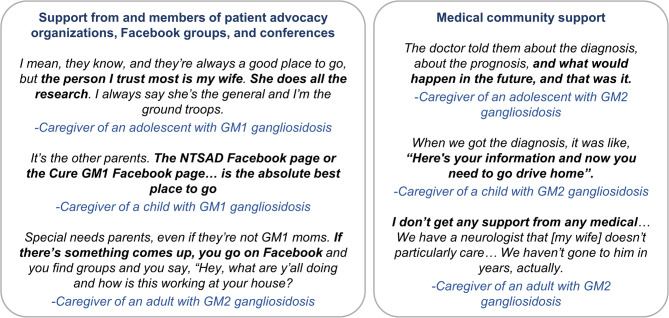
Resources available for the caregivers of individuals with GM1/GM2 gangliosidoses. NTSAD, National Tay–Sachs & Allied Diseases Association

Apart from the extended family, the caregivers mostly relied on patient advocacy organizations (PAOs), social media groups, and conferences as their primary sources of support (Fig. 4). The caregivers reported that they had also received support from the medical community, and some mentioned that their loved ones were being seen by excellent specialists who helped monitor the disease progression (Fig. 4).

### Resources/support needed by caregivers to assist individuals with GM1/GM2 gangliosidoses

The caregivers described the need of a range of resources and support, including education/awareness among the medical community, access to treatment and equipment, financial resources, and broader public support, to help reduce their caregiving burden (Table 5).

**Table 5:** Range of resources/disease-modifying treatments/broader public awareness to reduce caregivers’ burden

**Medical awareness**
*I would really like to see doctors and specialists who have better access*,* at least*,* to information about the diseases…so there would be a better repository of information about different aspects of the disease.* (Caregiver of an adolescent with GM1 gangliosidosis)
*I don’t get any support from any medical… We have a neurologist that [my wife] doesn’t* *particularly care for… We haven’t gone to him in years*,* actually.* (Caregiver of an adult with GM2 gangliosidosis)
**Equipment need**
*…the availability to equipment and things that help … There’s so many details in caring for these children*,* and equipment even as simple as a glove for your hand so that my child’s not biting at her hand or mine.* (Caregiver of a child with GM1 gangliosidosis)
*It would be really nice to not have to jump through hoops to get medical equipment. That would be phenomenal* (Caregiver of an adult with GM1 gangliosidosis)
**Treatment need**
*If there was anything*,* it would be a cure. But I know that’s not at the moment realistic.* (Caregiver of a child with unknown diagnosis)
*[Hoping] there might be something that would stop this disease and improve it a little bit…///…Yeah*,* prolong the progression…///…Stop the progress and improve it and I would feel then my life was not lived for nothing.* (Caregiver of an adult with GM2 gangliosidosis)
**Emotional support**
*Just having maybe therapists that specialize with children*,* complex children that we have*,* special needs children. To help you understand that it’ll be okay. Kind of navigate the different stages*,* the grieving process. Just having somebody to talk to that truly understands.* (Caregiver of a child with unknown diagnosis)
*Because every single day I’m overwhelming. I need to look at a psychologist therapy.* (Caregiver of an adolescent with GM1 gangliosidosis)
**Financial support**
*It’d be really great if I could get paid for my services taking care of the girls because we’ve lost the income of my employment.* (Caregiver of an adult with GM1 gangliosidosis)
*I’d be hoping the same as [REDACTED] mentioned [Get paid as a caregiver]. We can’t get paid just because we are parents. This is a big issue. We lost our job*,* we should be 24 h*,* but they said as a parent*,* we are not able to pay to you.* (Caregiver of an adult with GM2 gangliosidosis)
**Community awareness/services**
*It would be really great if there was a place that was safe that the girls could socialize at on a daily basis.* (Caregiver of an adult with GM1 gangliosidosis)
*I guess the community being more understanding of the physically challenged and taken more serious.* (Caregiver of an adult with GM2 gangliosidosis)
**Government support**
*Well*,* now that you say that*,* that would be something*,* in the state that I am in*,* if there could be financial help. If I could get paid to be his caregiver*,* that would be amazing. I don’t see why I can’t* (Caregiver of an adolescent with GM1 gangliosidosis)
*We went about 2 years ago to the city and back to get the approval to have ramp and special…we had problems with the city*,* that they said no*,* you can’t do it [put ramp and addition and other things].* (Caregiver of an adolescent with GM2 gangliosidosis)
**Respite requirement**
*It’s also just finding those people that are skilled that are quality that you can trust and that truly will do the things that are difficult to do for your child*,* and learn your child*,* and then keep them…those people.* (Caregiver of a child with GM1 gangliosidosis)
*Just somebody to take the physical load off. I never know when he’s going to fall. I don’t know when he’s going to drop something or I’m going to need to pick up something*,* carry his suitcases here and there.* (Caregiver of an adult with GM2 gangliosidosis)

For the child with an unknown diagnosis, attributing the quote to a specific participant was not possible given the qualitative nature of the study

The caregivers reported that the need for better awareness in the medical community would help alleviate some of the stress associated with caregiving burden, specifically through early diagnosis; for example, a caregiver of an adolescent with GM2 gangliosidosis stated:

*“It took 3 years to get that diagnosis. They’ve [the family] seen more than 10 neurologists.”* The caregivers required information and support regarding managing the disease from the medical community. A caregiver of an adolescent with GM2 gangliosidosis mentioned:

“The doctor told us about the diagnosis, about the prognosis, and what would happen in the future, and that was it.”

The caregivers also mentioned the necessity of better treatment access to avoid worsening of the disease and improve symptoms (Table 5). However, access to therapy was mentioned as one of the challenges of managing symptoms and care by the caregivers of adults with the disease. Often children and adolescents received a variety of therapies at school but for adults, these services could be more challenging to find and access. Two caregivers of adults with GM1 gangliosidosis described the challenges experienced in symptom care and management as:

“We don’t get any. When they went into the adult program, that was no longer an option. No PT, no OT, no nothing. . ///. . Yeah, that’s a thing that…if I have to talk to a GM1 family that has a younger kid, the best advice I give them is you get everything you need in line by the time they turn 18, because the world changes the morning they wake up and they’re 18. Society looks at the kids different. Society looks at you different. It’s like they’re not better, there’s no hope, see you later, bye. Everything becomes a fight. . ///. . .”

Some caregivers expressed the crucial need for access to medical equipment to perform caregiving activities, while others mentioned their desire for financial support or compensation programs (Table 5). Several participants mentioned that in the United States there are some states where caregivers have easier access to these compensation programs than others. A caregiver of an adolescent with GM1 gangliosidosis described:

“But to me, when you’re dealing with a disease like this and you have these extenuating circumstances, why are there not programs out there that are more easily accessible? Or a program at all where a financial burden can be lifted for parents like us?”

In addition, the caregivers requested psychological therapy due to emotional burden, increased community awareness, and support from other reliable caregivers to provide respite from all-encompassing caregiving responsibilities (Table 5). The caregivers expressed that they would welcome caregiving support from other individuals, but they were reluctant to let others provide it. This is in part due to distrust of others’ qualifications and the vulnerability of their loved ones.

A caregiver of an adult with GM2 gangliosidosis expressed:

“Honestly, just someone to lean on sometimes. I just don’t have that. I have my girls and I don’t want to put that on them, obviously. They’re young and have enough to deal with without that. I have myself. That is it.”

### Conceptual model of caregiving for individuals with GM1 and GM2 gangliosidoses

**Fig. 5:**
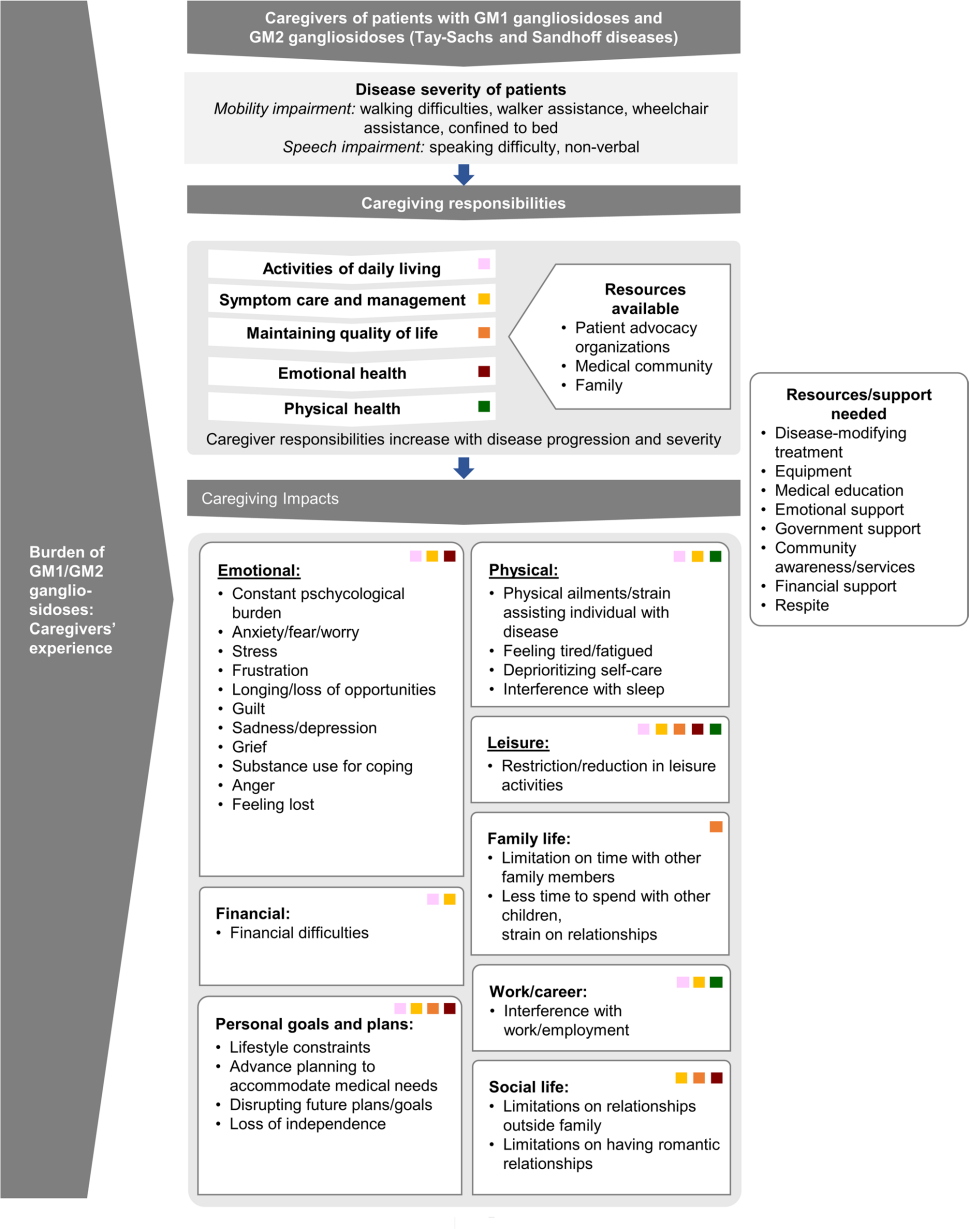
A conceptual model of GM1/GM2 gangliosidoses from caregivers’ perspective. The underlined concepts are the most reported top three categories of impacts among all others. The squares indicate a relationship between the particular responsibility (for example, physical health) and associated impacts (for example, physical, leisure, and work/career)

A conceptual model of GM1 and GM2 gangliosidoses caregiver burden developed using the concepts from focus groups identified five categories of responsibilities and 25 impacts (Fig. 5).

As an example of inter-relationships in the model, the caregivers reported that watching their loved ones lose the ability to move and communicate (mobility and speech impairment) had an emotional impact. A caregiver of an adolescent with GM2 gangliosidosis mentioned:

“You start getting depressed because you’re so stressed and angry. You’re angry because the disease is taking away your child and you can’t really do anything about it.”

According to the caregivers, assisting with ADLs had a physical impact on those who had to lift and move their loved ones. A caregiver of a child with GM2 gangliosidosis expressed:

“I have a bad back, and it’s from picking up [REDACTED] dead weight off the floor. They didn’t teach me [to lift properly].”

Another caregiver of an adolescent with GM2 gangliosidosis highlighted the physical strain due to caregiving:

“There’s physical fatigue. Our younger daughter, she isn’t walking anymore, and that’s been within the past year. She’s almost as tall as me, so it’s hard to move her around just because she’s so long.”

Moreover, elderly caregivers reported that they were unable to live the life they envisioned as a retiree due to the care they needed to provide to their loved ones. Ensuring physical health, such as helping with physical therapy exercises, was time consuming and limited the caregiver’s time to pursue other activities and hobbies. A caregiver of a child with GM1 gangliosidosis mentioned:

“Yeah, I usually get some exercise, but now I don’t do it anymore. I don’t have time, I think. I Usually maybe just like go to the park and stay there for some time."

Another caregiver of an adolescent with GM2 gangliosidosis emphasized:

“Because it’s so hard for us to get anywhere, to meet with anyone, to visit. If I go to my relative’s home out of state, if their house isn’t accessible, we can’t go. If the restaurant isn’t accessible, we can’t go."

Maintaining their loved ones’ QoL by providing companionship, entertainment and, in some cases, education also impacted the caregivers’ ability to spend time with other family members or friends, and their availability to pursue their own relationships. According to a caregiver of a child with GM1 gangliosiodis:

“I do also see in terms of friendships sometimes it’s difficult because I think many people are intimidated. They see the baggage you’re carrying. It’s visible, so it’s a lot to take on."

A caregiver of an adolescent with GM2 gangliosidosis mentioned:

“Family, friend. We realized that we are alone in this. Everyone told us that “whatever you need, I’m here,” but they knew that that really wasn’t the case. Other people don’t know how To act in the situation, so they walk away. And we really feel alone sometimes"'

## Discussion

The present qualitative study provides a holistic insight into the caregiving experience of those providing support to individuals living with GM1 and GM2 gangliosidoses throughout their life span. These findings highlight a significant and multifaceted burden experienced by caregivers, characterized by emotional, physical, leisure, family life, and financial strains. The burden persists, irrespective of the specific disease type or the age of the care recipient and intensifies as the caregivers assume greater responsibilities with disease progression. These findings also extend previous research reporting a significant burden, especially the emotional toll of GM1 gangliosidosis on the caregivers of children and adolescents [23].

A conceptual model was developed to capture the relationships among the severity of individuals being cared for, caregiving responsibilities, and the impacts of these responsibilities on the caregiver’s QoL (Fig. 5). The conceptual model illustrates the direct association of functional limitations (mobility and speech impairment) and caregiving responsibilities. Caregiver responsibilities increase with disease progression and severity, thereby inflicting impacts affecting every aspects of their lives; for example, managing activities of daily living imposes impacts, including emotional, leisure, physical, financial, work/career, and personal goals/plans. Similar impacts, as well as impacts on social life, were endured by caregivers while taking care and managing the symptoms of their loved ones. The conceptual model further explains that despite extensive support from PAOs, medical community, and their families, caregivers need a range of resources, including financial, educational, and medical support. This model can provide guidance for future research as a framework not only to understand the multifaceted burden of the caregivers, but also to design interventions that may improve experiences of individuals with GM1 and GM2 gangliosidoses and their caregivers.

The caregivers highlighted the importance of supportive therapies, such as physical, speech, and occupational therapies, as well as assistance with feeding needs and addressing the emotional and psychological impact of the disease, to primarily manage symptoms and improve the quality of life of their loved ones. Further, connecting with people in the GM1 and GM2 gangliosidoses communities enabled them to cope the challenges more effectively.

Not surprisingly, the findings of this study underscore the existence of several important unmet needs among caregivers of individuals with GM1 and GM2 gangliosidoses, encompassing primarily low disease awareness, lack of educational resources and therapeutic interventions to modify or halt disease progression, and supportive services to alleviate the burden of caregivers and their loved ones. Caregivers need to be appropriately counselled regarding the diagnosis, progression, expected outcomes, and anticipated complications of the disease. Moreover, increased awareness and education about the disease among caregivers could help them grapple with a range of debilitating symptoms. Appropriate genetic counseling should be offered to the parents of affected children who might be carriers and at risk of being carriers [24]. In contrast, PAOs emerge as vital partners of these families and sources of support, funding research, and raising awareness, with pivotal roles in building a sense of community by cultivating collaborations that can positively impact caregivers’ experiences. Nevertheless, there remain considerable opportunities for improvement, particularly in increasing awareness within the broader medical community and society at large.

Overall, the caregivers conveyed a strong need for a diverse range of resources to better manage the day-to-day responsibilities of caring for their loved ones. Assistance and awareness from the medical and broader social communities, as well as the government would also benefit caregivers and their loved ones, including access to specialists or physicians, with better information about the disease, counseling services, programs that can provide financial support and easier access to the existing ones, and most importantly, respite and care, thereby allowing them to take breaks from their caregiving responsibilities. With the intention of reducing the burden of caregiving, it is essential to recognize and address the challenges experienced by caregivers and provide them with the support they need to maintain their own health and well-being. With the caregivers’ experience varying greatly depending on the needs of the individual with the disease, familial circumstances, and support available, there is not a one-size-fits-all approach for supporting caregivers; in fact, in this study, many participants repeatedly stated that any resources would be welcomed.

The strength of this study lies in understanding the burden of GM1 and GM2 gangliosidoses from the perspective of caregivers and affirming the overall similarity of the burden experienced between the two diseases. Caring for individuals with rare diseases can have profound effects on the QoL of caregivers, as many experience physical, emotional, and financial strain due to the demands of caregiving [20, 22]. Overall, understanding the perspectives of caregivers can facilitate collaboration between researchers, healthcare providers, policy decision-makers and patient communities to drive progress in rare diseases research and care [22]. In the long term, these findings are an important contribution to the current understanding of caregivers’ experiences and may lead to greater overall awareness and the introduction of new interventions designed to better support individuals with the disease.

This study also has several limitations. Recruitment through PAOs networks may limit the generalizability of the findings to the broader population caring for individuals with GM1 and GM2 gangliosidoses. Owing to the fast-paced nature of group interviews, the individual source of comments in the transcripts could not always be identified. Moreover, the dynamic nature of the discussion did not allow all concepts to be consistently probed for each caregiver; this might have led to an underestimate of the number of impacts reported and/or the number of caregivers acknowledging certain impacts. The relatively small sample size of the caregivers of children or adolescents may limit the representativeness of the findings for these sub-populations. Additionally, during the evaluation of the relative importance of impact categories, some caregivers might have opted for either more or less than three top impacts had they not been limited to such a choice.

## Conclusion

The humanistic burden related to GM1 and GM2 gangliosidoses is substantial for caregivers, with long-term impacts. Caregiving responsibilities are diverse and can increase with disease progression but show little variability across caregivers of different age groups of individuals with the disease. The life of the caregiver is dominated by emotional, physical, leisure, family life, and financial burdens, regardless of the age of their loved ones and across GM1 and GM2 gangliosidoses. The findings of the conceptual model of caregiving burden may prove useful in understanding the impact of both GM1 and GM2 gangliosidoses. These findings can provide important insights to enhance clinical care and help assess the value of novel therapies while advocating for the resources needed to alleviate the burden and improve the lives of caregivers and their loved ones with GM1 and GM2 gangliosidoses.

## Supplementary Information

Below is the link to the electronic supplementary material.

Supplementary Material 1.

## Data Availability

All data generated or analyzed during this study are included in this article and its supplementary information files. Patient-level data will be anonymized and study documents will be redacted to protect the privacy of the participants.

## Abbreviations

ADLs: Activities of daily living
NTSAD: National Tay–Sachs and Allied Diseases Association
PAO: Patient advocacy organization
QoL: Quality of life

## Footnotes

1 PT, physical therapy; OT, occupational therapy.
