# Supplementary material for: Burden of caregiving of individuals with GM1 and GM2 gangliosidoses in the United States: a qualitative study
Source: Orphanet J Rare Dis. 2025 Nov 20;20:597. doi: 10.1186/s13023-025-04030-6 (PMC12632087; doi:10.1186/s13023-025-04030-6)
Supplement: Supplementary file 1 — Supplementary Material 1. [file 13023_2025_4030_MOESM1_ESM.docx]

**Supplementary Table 1** Concept saturation of impacts

| **Focus group** | **In-person** | | | **Online** | | | **Total** |
| --- | --- | --- | --- | --- | --- | --- | --- |
|  | **Based on age of individuals**  **with the disease** | | | **Based on age of individuals**  **with the disease** | | |  |
|  | **Adults** | **Adolescents** | **Children** | **Adults** | **Adolescents** | **Children** |  |
| **Number of participants^a^** | 6 | 3 | 5 | 7 | 6 | 2 | 29 |
| **Count of new concepts** | 20 | 4 | 1 | 0 | 0 | 0 | 25 |
| **Concepts first appearing in the group (%)** | 80 | 16 | 4 | 0 | 0 | 0 | 100 |
| **Concepts** | | | | | | | |
| Constant psychological burden | X |  |  |  |  |  |  |
| Anxiety/fear/worry | X |  |  |  |  |  |  |
| Financial difficulties | X |  |  |  |  |  |  |
| Physical ailments/strain | X |  |  |  |  |  |  |
| Limitations on time with other family members | X |  |  |  |  |  |  |
| Limitations on relationships outside of family | X |  |  |  |  |  |  |
| Restriction/reduction in leisure activities | X |  |  |  |  |  |  |
| Stress | X |  |  |  |  |  |  |
| Feeling tired/fatigued | X |  |  |  |  |  |  |
| Interference with work and employment | X |  |  |  |  |  |  |
| Deprioritizing self-care | X |  |  |  |  |  |  |
| Frustration | X |  |  |  |  |  |  |
| Longing/loss of opportunities | X |  |  |  |  |  |  |
| Interference with sleep | X |  |  |  |  |  |  |
| Constraints on lifestyle | X |  |  |  |  |  |  |
| Guilt | X |  |  |  |  |  |  |
| Limitations on the ability to have romantic relationships | X |  |  |  |  |  |  |
| Loss of independence | X |  |  |  |  |  |  |
| Substance use for coping | X |  |  |  |  |  |  |
| Anger | X |  |  |  |  |  |  |
| Sadness/depression |  | X |  |  |  |  |  |
| Planning in advance to accommodate medical needs |  | X |  |  |  |  |  |
| Disrupting future plans/goals |  | X |  |  |  |  |  |
| Feeling lost |  | X |  |  |  |  |  |
| Grief^b^ |  |  | X |  |  |  |  |

^a^The numbers are based on the count of caregivers who reported

Not all concepts or questions were consistently probed for each caregiver due to the nature of a focus group discussion

^b^This is related to what the loved one’s life has become/is becoming, loss of what their life could have been


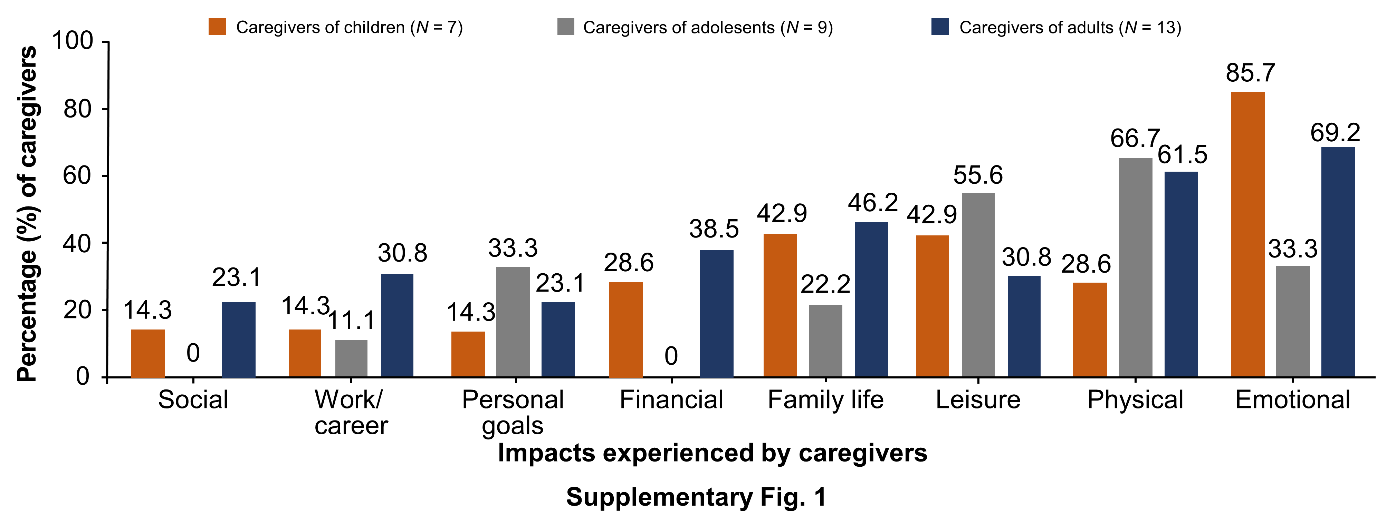


**Supplementary Fig. 1** Categories of impacts reported by caregivers. The numbers above the bars represent the percentage of individuals with the disease. N, total number of individuals with the disease
